# Mapping internal brainstem structures using T1 and T2 weighted 3T images

**DOI:** 10.3389/fnimg.2023.1324107

**Published:** 2023-12-15

**Authors:** Susanne G. Mueller

**Affiliations:** Department of Radiology, University of California, San Francisco, San Francisco, CA, United States

**Keywords:** brainstem, internal structures, segmentation, T1, T2, aging

## Abstract

**Background:**

Many neurodegenerative diseases affect the brainstem and often do so in an early stage. The overall goal of this project was (a) to develop a method to segment internal brainstem structures from T1 and T2 weighted sequences by taking advantage of the superior myelin contrast of the T1/T2 ratio image (RATIO) and (b) to test if this approach provides biological meaningful information by investigating the effects of aging on different brainstem gray matter structures.

**Methods:**

675 T1 and T2 weighted images were obtained from the Human Connectome Project Aging. The intensities of the T1 and T2 images were re-scaled and RATIO images calculated. The brainstem was isolated and k-means clustering used to identify five intensity clusters. Non-linear diffeomorphic mapping was used to warp the five intensity clusters in subject space into a common space to generate probabilistic group averages/priors that were used to inform the final probabilistic segmentations at the single subject level. The five clusters corresponded to five brainstem tissue types (two gray matters, two mixed gray/white, and 1 csf/tissue partial volume).

**Results:**

These cluster maps were used to calculate Jacobian determinant maps and the mean Jacobians of 48 brainstem gray matter structures extracted. Significant linear or quadratic age effects were found for all but five structures.

**Conclusions:**

These findings suggest that it is possible to obtain a biologically meaningful segmentation of internal brainstem structures from T1 and T2 weighted sequences using a fully automated segmentation procedure.

## 1 Introduction

The brainstem is the main gateway for information flow in and out of the cerebrum and plays a major role in locomotion, sensory processing, autonomic control, consciousness and even cognitive function. Many neurodegenerative diseases such as Alzheimer's and Parkinson's disease affect the brainstem and often do so in a relatively early or even prodromal stage (Winkler et al., 2011; Braak and Del Tredici, 2012; Seidel et al., 2015; Rüb et al., 2016). Consequently there is a considerable interest in assessing brainstem structure and function *in vivo*. However, except for the substantia nigra and nucleus (ncl.) ruber, brainstem nuclei and tracts are usually not discernible in MR images acquired for clinical purposes. This prompted the development of several dedicated brainstem sequences, e.g., high resolution and high field DTI and fMRI, MP2Rage for multi-contrast segmentation, 3D multi-echo FLASH sequence for multiparametric mapping, iron sensitive susceptibility mapping, myelin water fraction mapping and neuromelanin sensitive turbo-spin echo and magnetization transfer weighted sequences (Sasaki et al., 2006; Lambert et al., 2014; Faull et al., 2015; Betts et al., 2017; Keuken et al., 2017; Bianciardi et al., 2018; Priovoulos et al., 2018; Sclocco et al., 2018; Liu et al., 2019; Bouhrara et al., 2020; Mueller, 2020). These specialized sequences depict the targeted brainstem structures with impressive detail but time constraints and additional requirements such as for example ultra-high field magnets or need of expert post-processing make them difficult to implement in clinical protocols.

Whole brain T1 and T2 weighted sequences however are routinely acquired in clinical 3T brain imaging protocols. Their signal intensities strongly covary with myelin content but while the T1 signal increases with increasing myelin content, the T2 signal decreases. Glasser and Van Essen (2011) and Van Essen et al. (2013) exploited this divergent behavior by calculating a T1/T2 ratio image (RATIO) that has a greatly enhanced cortical myelin signal and is now commonly used for cortical myelin mapping. The brainstem also contains sparsely myelinated structures, e.g., brainstem nuclei or the spinothalamic tract, and densely myelinated/ iron rich structures, e.g., the cortico-spinal tract or the nucleus ruber. Densely and sparsely myelinated structures are tightly packed together resulting in steep myelination gradients similar to those observed in the cortex. Therefore, the RATIO image alone or in combination with the T1 and T2 weighted image could also be useful for the segmentation of internal brainstem structures. The first aim of this study was to test this assumption using a modification of a brainstem segmentation approach based on MP2Rage derived T1 weighted and T1 relaxation images (Mueller, 2020). The second aim was to test if this approach provides biological meaningful information by investigating the effects of typical aging on the volumes of the different brainstem gray matter structures identified in these segmentations.

## 2 Methods

### 2.1 Population

The imaging data of 675 subjects [age mean (SD): 58.9 (14.9), age range: 36–100, m/f: 292/382] from the Human Connectome Project Aging (HCA) was used for this project. The aim of the HCA is to study “typical aging,” i.e., its population includes participants who exhibit health issues typically seen in their age cohort, e.g., hypertension, musculoskeletal pain, but do not suffer from pathological conditions, e.g., major depression, sleep apnea, stroke or suspected Alzheimer's disease etc. It collects a variety of structural and functional MR images as well as behavioral and biological data.

### 2.2 Imaging

All participants were scanned on a customized Siemens 3T “Connectome Skyra” at Washington University using a standard 32-channel Siemens receive and a body transmission coil (van Essen). The distortion corrected T1 weighted images [3D MPRAGE TR = 2400 ms, TE = 2.14 ms, TI = 1000 ms, FA = 8°, Bandwidth (BW) = 210 Hz per pixel, Echo Spacing (ES) = 7.6 ms, 0.8 mm isotropic resolution] and T2 weighted images (SPACE, TR = 3200 ms, TE = 565 ms, 0.8mm isotropic resolution with same matrix and slices as T1 weighted images) were used for this project.

### 2.3 Image processing

**Figure 1:**
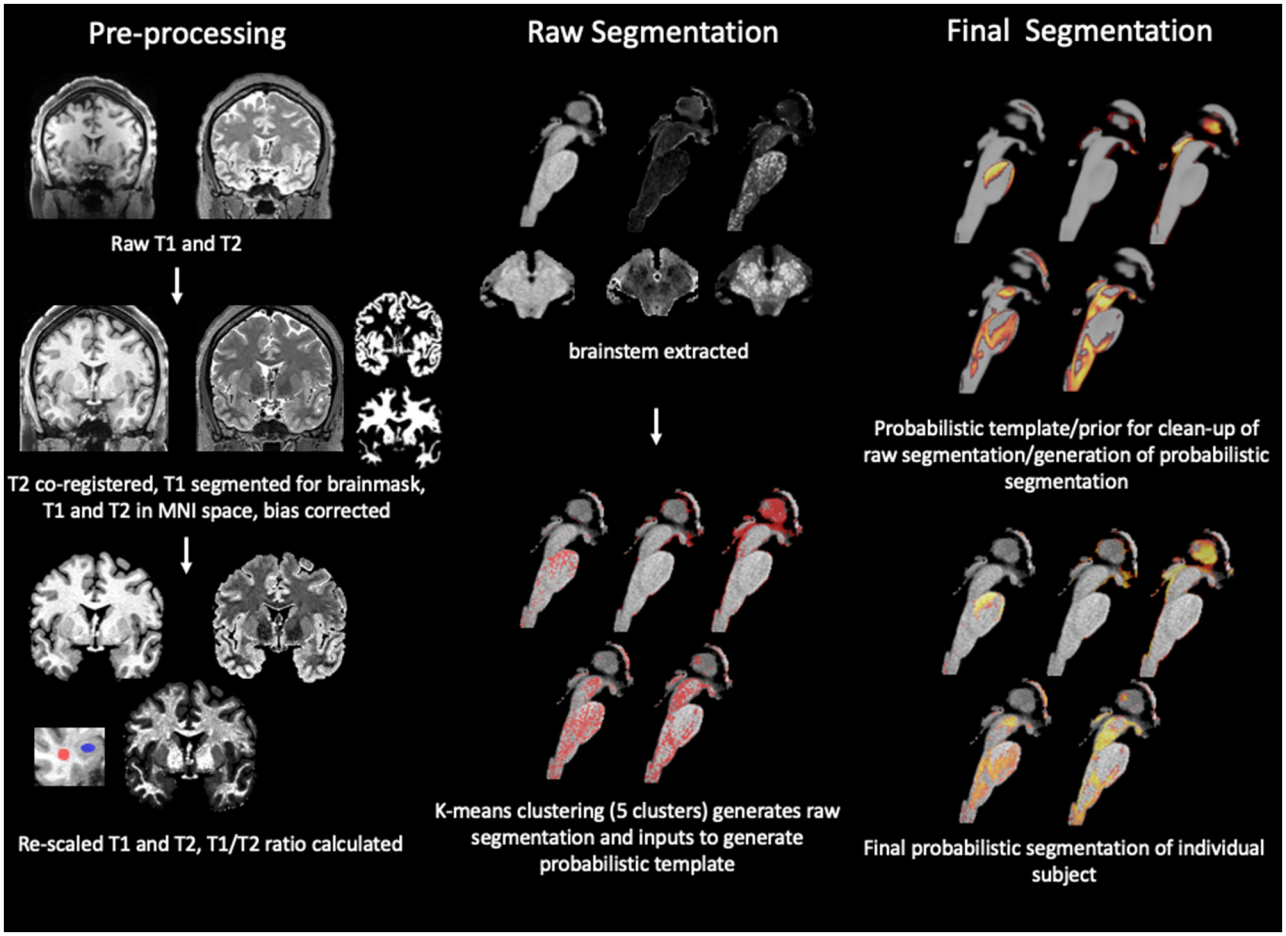
Summary of brainstem segmentation pipeline. The pipeline consists of 3 main modules of which each encompasses several steps. The first module is “pre-processing” that uses SPM12 routines for tissue segmentation with inbuilt additional bias correction and brainmask generation, followed by spatial normalization to the MNI space while maintaining the original image resolution. The T1 and T2 image are re-scaled and the T1/T2 or RATIO image calculated. The rois used to extract the gray (blue) and white (red) matter intensities reported in Table 2 are shown in the insert. The images are then passed on to the second module whose first step is to use a binary brainstem/thalamus mask in MNI space to extract the brainstem/diencephalon from each of the three images. The next step uses a k-mean clustering algorithm to identify 5 intensity clusters. The cluster labels are converted into an image in subject space as binary first pass segmentations. This is followed by the generation of a group average probability map or prior map for each cluster by warping the first pass binary segmentations into a common space using SPM's DARTEL “create template algorithm” which is also the first step of the last module or “final segmentation,” i.e., the generation of probabilistic group averages to be used as priors to refine the segmentation outputs. The transformation matrix from this step was inverted and used to warp the probabilistic group averages into the subject/MNI space. The information from the priors was combined with the distance information from the clustering step which allowed to clean-up voxels assigned to a cluster not consistent with the probability information and to convert the binary first pass segmentation into a probabilistic final segmentation. Please see “Methods” for more details.

The work flow is summarized in Figure 1. The segmentation of the internal brainstem structures used a modification of the MP2Rage based approach (Mueller, 2020). The T2 SPACE image (T2) was co-registered to MPrage T1 weighted image (T1). In the next step, the bias correction algorithm implemented in SPM12's “unified segmentation” (https://www.fil.ion.ucl.ac.uk/spm/software/) was used to generate bias corrected versions of the T1 and co-registered T2 (rT2) and to obtain gray and white matter tissue maps from the T1. The gray matter map was spatially normalized into the MNI space using SPM12's “normalize” function and template, and the forward and inverse transformations of this step calculated. The former was applied to all outputs (T1, rT2, tissue maps, and the binary brain tissue mask that was generated by combining gray and white matter maps thresholded at 0.2). The next step was to enhance the gray/white contrasts of the T1 and rT2 images using a modification of the linear scaling procedure proposed by Ganzetti et al. (2014), i.e., instead of obtaining reference intensities from non-brain tissue regions it used fixed, experimentally determined reference intensities at Ref1 = 100 and Ref2 = 20. The CSF map and the white matter tissue map were thresholded at 0.9 to identify voxels with high probability to be either CSF voxels or white matter voxels. The intensities of these high probability CSF and white matter voxels were extracted from each subject's T1 and rT2 and the modes of their intensity histograms determined after excluding voxels corresponding to vessels whose intensity was either below the 1th percentile (rT2) or above the 99th percentile (T1). The histogram modes were used to re-scale the T1 and rT2 using the following formulas:

T1:


$$\begin{array}{l} T1fact\;=\;abs(\frac{Ref1-Ref2}{wm_{T1_{Mode}}-csf_{T1_{Mode}}})\\ T1shift\;=\;abs(\frac{{\left((wm_{T1_{Mode}}Ref2)-(csf_{T1_{Mode}}Ref1)\right)}^{*}2}{wm_{T1_{Mode}}-csf_{T1_{Mode}}})\\ \;T1_{cal}\;=\;(T1^{*}T1fact)-T1shift \end{array}$$

wm_T1_Mode, mode of histogram from voxels with more than 90% probability to be white matter, csf_T1_Mode, mode of histogram from voxels with more than 90% probability to be CSF voxels, T1fact, scale factor for T1 by which the original intensity range is reduced, T1shift, distance by which the intensity histogram is moved toward the left, abs, absolute. T1_cal, re-scaled T1 image.

rT2:


$$\begin{array}{l} rT2fact\;=\;(abs(\frac{Ref2-Ref1}{wm_{rT2_{Mode}}-csf_{rT2_{Mode}}}))\\ rT2shift\;=\;abs(\frac{\left(csf_{rT1_{mode}}Ref2)-(wm_{rT2_{Mode}}Ref1\right)}{wm_{rT2_{Mode}}-csf_{rT2_{Mode}}})\\ \;rT2cal\;=\;(rT2^{*}rT2fact)-rT2shift \end{array}$$

wm_rT2_Mode, mode of histogram from voxels with more than 90% probability to be white matter, csf_rT2_Mode, mode of histogram from voxels with more than 90% probability to be CSF voxels, rT2fact, scale factor for rT2 by which the original intensity range is reduced, rT2shift, distance by which the intensity histogram is moved toward the left, abs, absolute, rT2_cal, re-scaled rT2_map image.

The re-scaling introduced brain tissue voxels with negative intensities in the T1 and rT2. These negative voxels were identified and replaced with the mean of the intensities of non-negative first-order neighborhood voxels. The next step was to calculate the RATIO image from the re-scaled T1 and rT2. Voxels in the RATIO image whose intensity exceeded the 99th percentile were replaced with the mean of the intensities of the first-order neighborhood voxels with intensities at or below the 99th percentile. Combining the re-scaled T1 and rT2 in this way further increased the gray/white matter contrast. Table 1 summarizes the recalibration effects in 10 randomly selected data sets.

**Table 1:** Effects of rescaling on gray/white intensities.

**Image**	**Orig [mean(SD)]**	**Rescaled [mean(SD)]**
T1	1.35 (0.04)#	1.42 (0.10)^*^
T2	1.45 (0.13)#	1.93 (0.31)^*^
RATIO	2.88 (0.52)	

Gray matter intensity, mean intensities extracted from 61 voxels in left amygdala; white matter intensity, mean intensities extracted from 69 voxels in deep left temporal lobe white matter. The intensities were used to calculate white/gray ratios for the original and rescaled T1 image and to calculate gray/white ratios for the original and rescaled T2 images from 10 randomly selected subjects. The white/gray (T1) and gray/white (T2) ratios of the original images were significantly smaller than those of the rescaled images (#gray/white ratio of orig smaller than rescaled p < 0.05 with two-tailed t-tests). The intensities extracted with the same rois from T1/T2 ratio image (RATIO) calculated from the rescaled images were higher than the intensities ratios from the rescaled T1 and T2 images (^*^gray/white ratio of RATIO higher than gray/white ratios of rescaled p < 0.05, two-tailed t-tests).

### 2.4 First-pass binary brainstem segmentation and prior generation

A brainstem/thalamus label in MNI space generated from the 2009 ICBM 152 T1 atlas was used to extract the brainstem/thalamus images (bs) from the re-scaled whole brain T1_cal, rT2_cal and RATIO images. The brainstem/thalamus T1_cal image was thresholded to generate a subject-specific binary brainstem tissue mask. This mask was used to extract the tissue intensities from each subject's brainstem T1_cal, rT2_cal and RATIO image. The intensities from each image type were converted into z-scores that were supplied to the k-means clustering algorithm implemented in Matlab 9.4 (The Math Works, Natick, MA) (number of clusters *n* = 5, squared Euclidian distance function, maximum number of iterations = 1000, replicates = 100). The optimal number of clusters *n* = 5 had been determined experimentally by exploring the range from 4 [no. of tissue components identified by Lambert et al. (2014)] to 8 in three subjects. With *n* = 5 clusters, one of the resulting first-pass cluster images corresponded to the outer brainstem boundaries and the remainder highlighted different internal brainstem structures when displayed in image space. The cluster centroid information of each subject was matched to the centroid information of a randomly selected reference subject and the cluster numbering accordingly changed to ensure a consistent cluster numbering/centroid assignment across different subjects.

### 2.5 Evaluation of segmentation performance

The rationale for starting out by entering all three images into the clustering algorithm was based on the following reasoning: A white matter voxel in the T1_cal whose intensity falls into the gray matter range due to noise is assigned to the same cluster as a gray matter voxel in a gray matter structure. Since noise is random, the likelihood that this voxel has also a gray matter intensity in the T2_cal image is low and this also mitigates the influence of the T1 noise on the signal intensity in the RATIO image. Combining all three images therefore increases the likelihood that this white matter voxel is either assigned to the correct cluster or at least has a larger distance to the gray matter cluster centroid than it would have if the segmentation would be based on the T1_cal alone. The next step was to investigate if this assumption is indeed true. This was done by obtaining raw segmentations using each of the three images as the sole input and combinations, i.e., RATIO and T2, T1 and T2. The following three indices were calculated for the three image input and each clustering variant: (1) Misclassification index: Percentage of voxels assigned to a cluster that fall outside its probabilistic group average (thresholded at 0.3, see next paragraph) averaged over all 5 clusters. A low value indicates good clustering performance. (2) Misclassification distance: The standardized centroid distance (see next paragraph for definition) of all misclassified voxels as defined in 1 averaged over all 5 clusters. A low value indicates a good clustering. (3) Subthreshold silhouette index. The silhouette coefficient (range −1 to 1 with higher values indicating a better performance) is a commonly used method to assess the clustering performance. It provides an excellent measure to assess clustering performance based on voxel intensity but is oblivious to spatial information, i.e., the hypothetical noise affected white matter voxel would receive a similar silhouette coefficient as a true gray matter voxel in a gray matter structure if the segmentation is solely based on the T1_cal intensity. Adding the information from the T2_cal and RATIO image decreases the silhouette coefficient of the noise affected white matter voxel or “downgrades” its membership to the gray matter cluster but leaves the silhouette coefficient of the true gray matter voxel mostly unchanged thus confirming its membership to this cluster. “Downgrading” noise-affected voxels in this way will increase the number of voxels whose silhouette coefficient falls below 0.6. A segmentation that successfully “downgrades” misclassified voxels is expected to have a high “subthreshold silhouette index.” Taken together, the best performing segmentation is expected to be characterized by a low misclassification index, a low mean misclassification distance and a high subthreshold silhouette index.

### 2.6 Final segmentation

The sorted first-pass cluster images from all 675 subjects were used as inputs for DARTEL's create template algorithm in SPM12 to generate a probabilistic 5 cluster population template. The transformation matrices generated for each subject during this process were inverted and applied to the group average of each cluster to project the latter into each subject's cluster image space. Using the information from the probabilistic group averages in subject space and the standardized centroid distances (original distances transformed to values between 0 and 1 with voxel closest to the cluster centroid = 1 and the voxel with greatest distance= 0), each brainstem voxel in the individual subject was assessed for consistency. It was considered consistent if the cluster assignment based on centroid distance coincides with the cluster assignment based on probabilistic group averages, i.e., its probability to belong to this cluster is higher than that to belong to one of the other clusters. Voxels with inconsistent assignments were re-assigned to the cluster suggested by the probabilistic group averages (alternate cluster) if they met one of the following conditions. (1) Probability that voxel belongs to the alternate cluster is ≥0.75 (2) Probability that voxel belongs to the alternate cluster is higher by ≥0.20 than probability to belong to original cluster. Finally, the binary cluster images for each subject were converted into probabilistic cluster images by multiplying them with the corresponding probabilistic group average weighted by the standardized centroid distance information for this subject.

### 2.7 Image analysis

The probabilistic cluster images in MNI space from all 675 subjects were used as inputs for DARTEL's create template algorithm in SPM12 to generate the final 5 cluster population template. On these templates 48 brainstem regions of interest (roi) (periaqueductal gray (PAG), ventral tegmental area (VTA), rostromedial tegmental (Trm) and laterodorsal tegmental (Tld) nucleus (ncl), raphe dorsalis ncl. (DR) median raphe ncl. (MedR), raphe magnus ncl. (MR), raphe obscurus (OR) and raphe pallidus ncl. (PR), left and right substantia nigra (SN), ncl. ruber (NR), ncl. pedunculopontinus (PP), ncl. reticularis cuneiformis (CR), ncl. reticularis pontis oralis (RPO), ncl. reticularis pontis tegmenti (RPT), ncl. reticularis pontis caudalis (RPC), ncl. reticularis gigantocellularis and parvocellularis (RG), medullary reticular ncl. or ncl. reticularis medullae oblongatae (RMO), locus coeruleus (LC), ncl. subcoeruleus (SC), ncl. parabrachialis (PB), ncl. pontis (PN), ncl. tractus solitarii (NTS), ncl. olivarius inferior (OI), ncl. ventrolateral medulla (VLM), parafacial zone (PZ), colliculus superior (CS), and colliculus inferior (CI) were identified using the brainstem atlases from Naidich et al. (2009) and Paxinos and Huang (2011) as references and manually delineated. The transformation matrices generated during the final 5 cluster population template building were converted into Jacobian determinant maps from which the mean intensities from each of the 48 rois were extracted for each subject.

### 2.8 Statistical analysis

ANOVA tests with Scheffe *post-hoc* tests were used to compare misclassification indices, misclassification distances and silhouette indices of the different clustering variants. Linear and quadratic regression analyses with the mean Jacobian determinant of each roi as dependent and age (linear regression) and age and age squared (quadratic regression) as independent variable(s) were used to investigate the influence of age on each roi. False discovery rate (FDR) with *q* = 0.05 was used to correct for multiple comparisons.

## 3 Results

### 3.1 Segmentation performance

Please see also Table 2. As stated in Section 2.5, the best performing segmentation is characterized by a low misclassification index, a low mean misclassification distance and a high subthreshold silhouette index. The three image approach fulfills these criteria well given its low misclassification index and misclassification distance combined with the high subthreshold silhouette index. The segmentation based the combined information from the T1_cal and T2_cal image performs similarly well or slightly better as the three image approach regarding misclassification distance and high subthreshold silhouette index. However, its misclassification index is almost twice that of the three image approach. This indicates that although the T1 and T2 combination is effective downgrading misclassified voxels, the three image approach creates fewer misclassified voxels. Despite its strong gray/white contrast, the RATIO image as sole input performed worse than the T1_cal or T2_cal image as sole inputs.

**Table 2:** Segmentation performance summary.

**k-mean clustering inputs**	**Misclassification index**	**Misclassification standardized distance**	**Silhouette index**
All 3 images	0.22 (0.03)	0.987 (0.002)	0.53 (0.03)
RATIO only	0.51 (0.04)^*^	0.996 (0.001)^*^	0.27 (0.01)^*^
T1 only	0.37 (0.03)^*^	0.994 (0.001)^*^	0.29 (0.00)^*^
T2 only	0.31 (0.05)^*^	0.993 (0.001)^*^	0.31 (0.01)^*^
RATIO & T2	0.28 (0.04)^*^	0.993 (0.001)^*^	0.31 (0.01)^*^
T1 & T2	0.43 (0.09)^*^	0.986 (0.002)	0.55 (0.01)^*^

^*^Different from “all 3 images” p < 0.05.

### 3.2 Tissue types in probabilistic population cluster maps or priors

**Figure 2:**
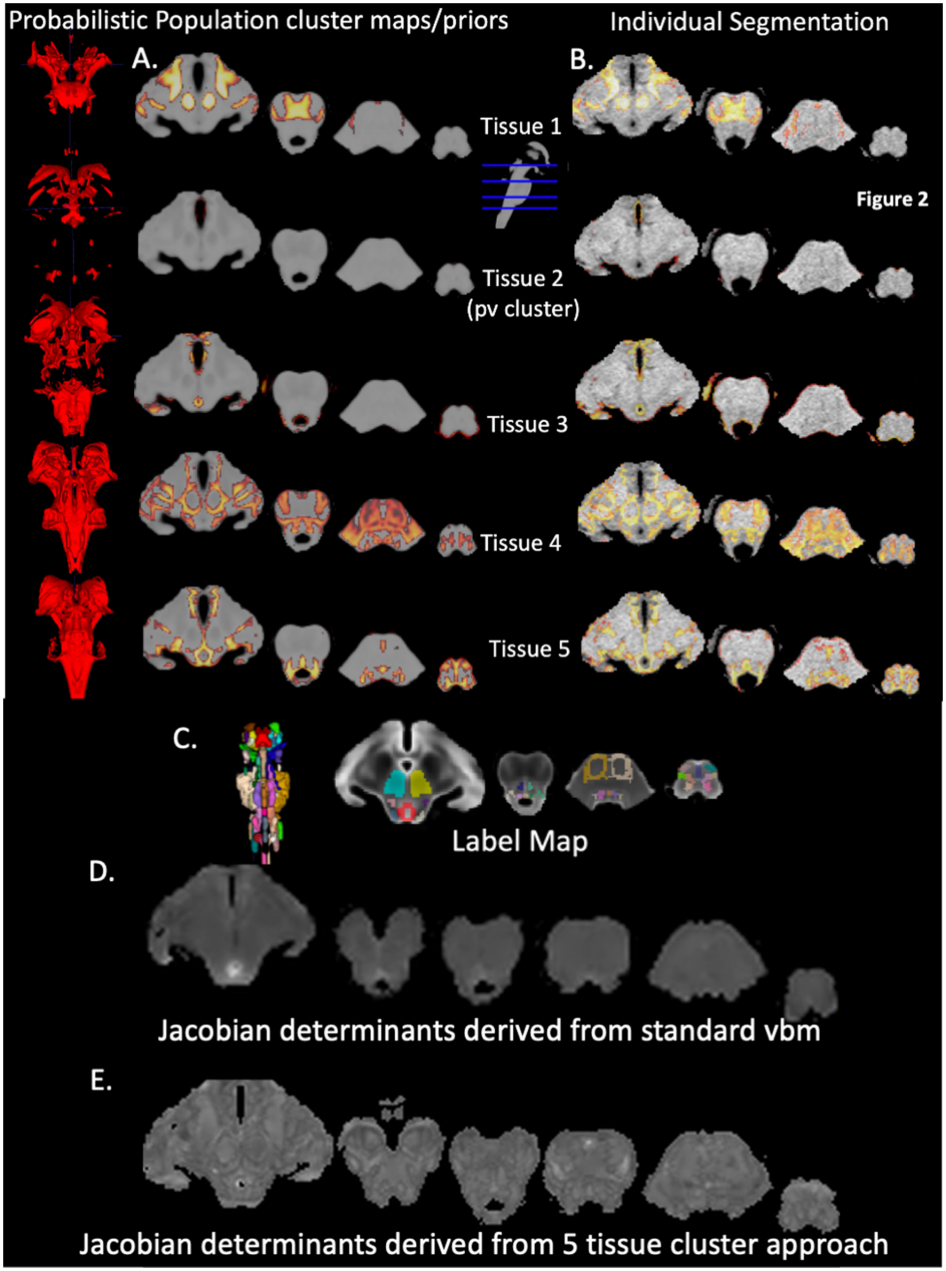
Brainstem segmentation outputs. The first 5 left-sided rows are 3D renderings and the corresponding projections of the 5 probabilistic population cluster maps or priors onto a population T1 brainstem image that were generated with DARTEL **(A)** The first 5 right sided rows show the final segmentations of an individual subject **(B)** The row below depicts a 3D rendering of the brainstem labels and the labels overlaid on the population T2 brainstem image **(C)** Below **(D)** is a Jacobian Determinant map generated by the standard VBM approach (input whole brain gray and white tissue maps) whereas the bottom row **(E)** shows the Jacobian Determinant map generated by supplying the 5 final segmentations of an individual to DARTEL. Details of individual structures, e.g. RN, NTS etc. are identifiable.

Please see also Figure 2, that depicts the probabilistic priors for each of the five clusters on the left side and an example of the quality of the final segmentation in a single subject on the right side. Using the brainstem atlas of Naidich et al. (2009) as reference, Cluster 1 encompasses mostly the iron rich SN (reticulata) and NR and white matter structures such as the frontopontine and parietotemporopontine tract and at the level of the pons sections of the middle cerebral peduncle. Cluster 2 is a “partial volume” cluster that consists of voxels at the brainstem CSF/tissue boundary. Cluster 3 is a gray matter cluster containing the PAG. Cluster 4 is a mixed gray/white cluster that besides gray/white transitions/partial volume predominantly contains white matter structures, e.g., superior and inferior cerebellar peduncles, corticospinal tract, spinothalamic tract, medial and lateral lemniscus, central tegmental tract but also gray matter structures, e.g., SN (compacta), CS, CI, PB, RPO, RPC, Tld, RG. Cluster 5 is a predominantly gray matter cluster that encompasses for example, VTA, CR, DR, LC, SC, MedR, MR, OI, NTS, PR, OR.

### 3.3 Regression analysis

**Figure 3:**
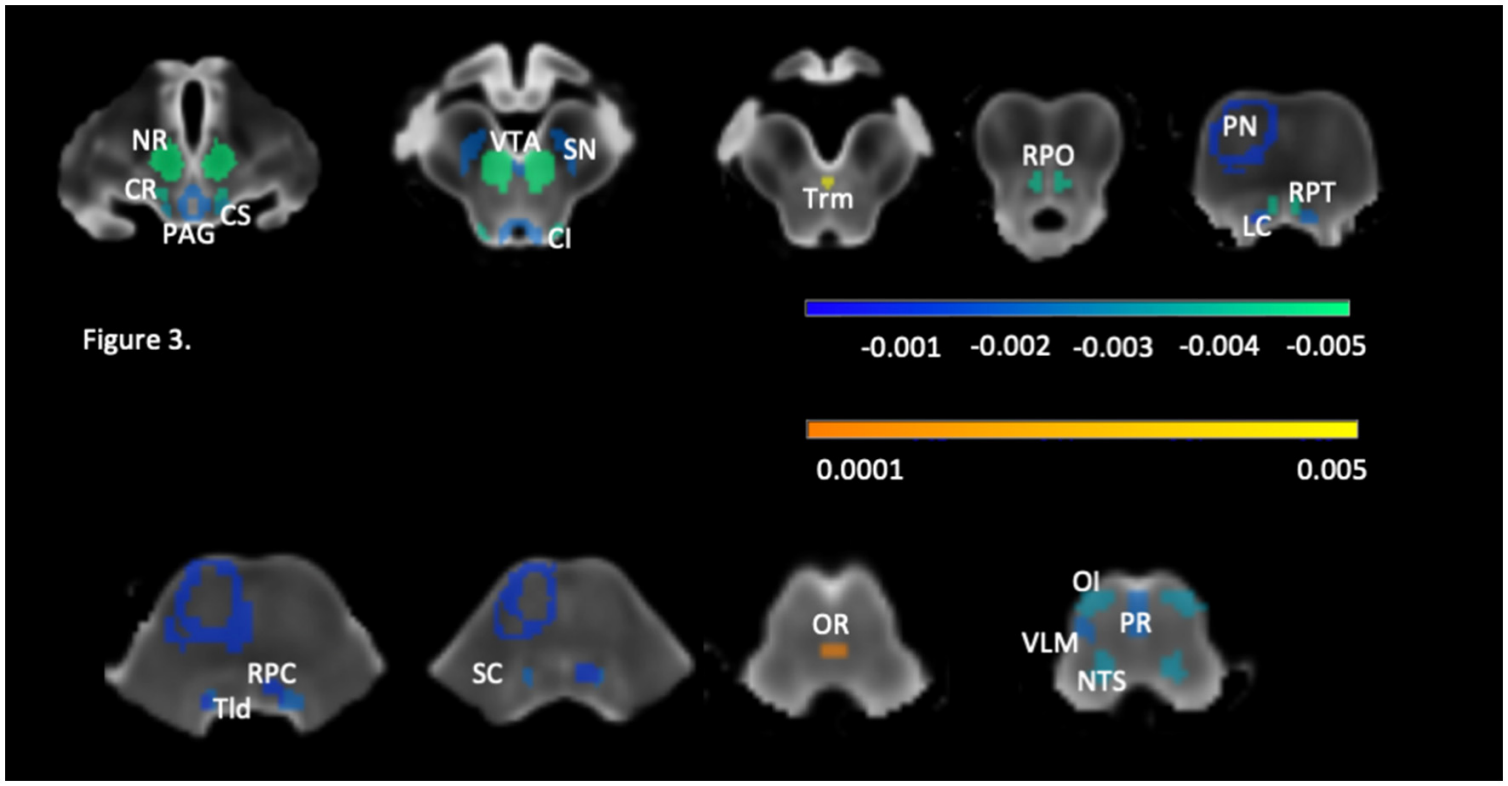
Brainstem structures with significant age effect after correction for multiple comparisons with (FDR, *q* = 0.05). The roi color reflects the regression coefficient strength (please see also Table 3), negative coefficients are indicated in cold and positive coefficients in warm colors. CI, colliculus inferior; CR, ncl. reticularis cuneiformis; CS, colliculus superior; LC, locus coeruleus; NR, ncl. ruber; NTS, ncl. tractus solitarii; OI, ncl. olivarius interior; OR, ncl. raphe obscurus; PAG, periaqueductal gray; PN, pontine nuclei; PR, ncl. raphe pallidus; RPC, ncl., reticularis pontis caudalis; RPO, ncl. reticularis pontis oralis, RPT, ncl. reticularis pontis tegmenti; SC, ncl. subcoeruleus; SN, substantia nigra; Tld, ncl. tegmentalis laterodorsalis; Trm, ncl. tegmentalis rostromedialis; VLM, ventrolateral medulla; VTA, ventral tegmental area.

Please see Table 3 and Figure 3. Significant negative linear associations between age and volume surviving FDR correction were found for 31 brainstem rois (PAG, VTA, NTS, SN, NR, OI, CS, CI, Trm, Tld, LC, SC, PR, CR, RPO, RPT, left PN, left VLM, and right RPC). The adjusted r squares ranged from <0.03 to 0.22. Age explained 20% or more of the variations of the volumes of the left and right NR and CR and more than 10% of the variation of the volumes of the left and right SN, RPO, OI and NTS. Negative significant associations that did not survive FDR correction were found for 10 additional brainstem rois (PFZ, RG, MedR, MR, right PN, right VLM, left RPC, and left RMO). Significant quadratic age effects surviving FDR correction were found for right SN, right RPT and OR. Of these, the OR was the only structure that showed only quadratic age effects. Quadratic age effects not surviving FDR correction were found for left SN, left CR, left RPT, right RG and bilateral VLM and RMO. Age had no significant effects on the volumes of the DR, left and right PP and PB.

**Table 3:** Results of linear and quadratic regression analyses.

**Group**	**Structure**	**Linear regression age coefficient**	**R square adjust**	***p*-value**	**FDR**	**Quadratic regression age coefficient quad**	**R square adjust**	***p*-value**	**FDR**
Autonomic	PAG	−0.002	0.035	0	Sig	0	0.033	0.915	Not sig
	L PB	0	−0.001	0.744	Not sig	0	−0.003	0.755	Not sig
	R PB	0	−0.001	0.837	Not sig	0	−0.002	0.452	Not sig
	L NTS	−0.003	0.187	0	Sig	0	0.186	0.914	Not sig
	R NTS	−0.003	0.164	0	Sig	0	0.164	0.423	Not sig
	L PFZ	−0.001	0.022	0	Not sig	0	0.023	0.141	Not sig
	R PFZ	−0.001	0.025	0	Not sig	0	0.027	0.15	Not sig
	L VLM	−0.002	0.031	0	Sig	0	0.036	0.041	Not sig
	R VLM	−0.002	0.023	0	Not sig	0	0.028	0.038	Not sig
Nigra	L SN	−0.002	0.135	0	Sig	0	0.139	0.045	Not sig
	R SN	−0.002	0.119	0	Sig	0	0.13	0.003	Sig
Pre-cerebellar	L NR	−0.006	0.195	0	Sig	0	0.196	0.209	Not sig
	R NR	−0.006	0.222	0	Sig	0	0.223	0.174	Not sig
	L PN	−0.001	0.032	0	Sig	0	0.036	0.066	Not sig
	R PN	−0.001	0.024	0	Not sig	0	0.023	0.525	Not sig
	L OI	−0.003	0.152	0	Sig	0	0.153	0.27	Not sig
	R OI	−0.003	0.161	0	Sig	0	0.161	0.345	Not sig
Sensory	L CS	−0.003	0.048	0	Sig	0	0.047	0.869	Not sig
	R CS	−0.003	0.084	0	Sig	0	0.086	0.121	Not sig
	L CI	−0.004	0.058	0	Sig	0	0.059	0.155	Not sig
	R CI	−0.004	0.078	0	Sig	0	0.079	0.189	Not sig
Tegmental	VTA	−0.002	0.114	0	Sig	0	0.011	0.826	Not sig
	Trm	0.005	0.056	0	Sig	0	0.064	0.164	Not sig
	L PP	−0.001	0.002	0.102	Not sig	0	0.001	0.849	Not sig
	R PP	0	−0.001	0.836	Not sig	0	−0.001	0.27	Not sig
	L Tld	−0.002	0.061	0	Sig	0	0.06	0.391	Not sig
	R Tld	−0.002	0.074	0	Sig	0	0.08	0.021	Not sig
Coeruleus	L LC	−0.001	0.058	0	Sig	0	0.057	0.544	Not sig
	R LC	−0.002	0.08	0	Sig	0	0.082	0.138	Not sig
	L SC	−0.002	0.047	0	Sig	0	0.046	0.446	Not sig
	R SC	−0.002	0.091	0	Sig	0	0.092	0.302	Not sig
Raphe	DR	0	0.001	0.245	Not sig	0	−0.001	0.817	Not sig
	MedR	−0.001	0.02	0	Not sig	0	0.021	0.258	Not sig
	MR	−0.002	0.023	0	Not sig	0	0.023	0.242	Not sig
	PR	−0.002	0.03	0	Sig	0	0.028	0.768	Not sig
	OR	−0.001	0.004	0.059	Not sig	0	0.014	0.006	Sig
Reticular	L CR	−0.004	0.203	0	Sig	0	0.207	0.035	Not sig
	R CR	−0.004	0.218	0	Sig	0	0.218	0.179	Not sig
	L RPO	−0.004	0.118	0	Sig	0	0.118	0.442	Not sig
	R RPO	−0.004	0.145	0	Sig	0	0.146	0.215	Not sig
	L RPT	−0.004	0.048	0	Sig	0	0.052	0.043	Not sig
	R RPT	−0.004	0.052	0	Sig	0	0.063	0.003	Sig
	L RPC	−0.001	0.027	0	Not sig	0	0.026	0.344	Not sig
	R RPC	−0.001	0.03	0	Sig	0	0.032	0.097	Not sig
	L RG	−0.001	0.004	0.05	Not sig	0	0.008	0.052	Not sig
	R RG	−0.001	0.005	0.038	Not sig	0	0.009	0.049	Not sig
	L RMO	−0.002	0.01	0.005	Not sig	0	0.017	0.019	Not sig
	R RMO	−0.001	0.002	0.114	Not sig	0	0.011	0.009	Not sig

PAG, periaqueductal gray, PB, ncl. parabrachialis, NTS, ncl. tractus solitarii, PFZ, parafacial zone; VLM, ventrolateral medulla: SN, substantia nigra; NR, ncl. ruber: PN, pontine nuclei; OI, ncl olivarius inferior, CS, ncl. colliculus superior; CI, nucleus colliculus inferior; VTA, ventral tegmental area; Trm, ncl tegmentalis rostromedialis;PP, ncl. pedunculopontinus; Tld; ncl. tegmentalis laterodorsalis, LC, locus coeruleus; SC, ncl subcoeruleus; DR, ncl. raphe dorsalis, MedR, ncl raphe medianus; MR; ncl raphe magnus; PR, ncl raphe pallidus; OR, ncl raphe obscurus; CR, ncl. reticularis cuneiformis; RPO, ncl. reticularis pontis oralis; RPT, ncl. reticularis pontis tegmenti; RPC, ncl reticularis pontis caudalis; RG; ncl. reticularis gigantocellularis; RMO, ncl. reticularis medullae oblongatae, adjust, adjusted.

## 4 Discussion

The study had the following main findings: (1) The myelin-sensitive RATIO image together with the re-scaled and thus contrast-enhanced T1 and T2 images used to calculate it allows to identify 5 tissue clusters depicting internal gray and white matter brainstem structures when supplied to k-means clustering. These 5 tissue clusters allow to generate probabilistic priors at the group level that together with the distance information from the clustering step can be used to eliminate misclassified voxels and to generate probabilistic brainstem tissue maps for individual subjects. In combination with a labeled brainstem atlas, these tissue maps can be used to obtain volumetric information of brainstem structures of interest for example by calculating Jacobian determinant maps. (2) The ability of the new brainstem segmentation approach to detect biological meaningful information was demonstrated by investigating the impact of typical aging on the volumes of 48 brainstem structures in 675 subjects from the HCA data repository. With the exception of DR, PP and PB whose volumes were not influenced by age, strong aging effects, i.e., typical aging explained at least 10% or more of the volume variability, were found for VTA, NR, CR, SN, RPO, OI and NTS. Effects smaller than 10% were found for the volumes of many of the remaining brainstem structures. The next paragraphs will discuss these findings in more detail.

To the best of our knowledge the T1/T2 brainstem segmentation method presented here is the first that uses sequences that are routinely acquired in clinical exams. The same sequences are also often used in research studies investigating large populations of healthy subjects such as the Human Connectome Project which allows to establish normal ranges. Furthermore, the process is fully automated and does not require expert user input. This indicates that this technique could potentially be used in clinical settings to probe for brainstem pathologies if the T1 and T2 sequences have the same and high enough resolution (ideally <1 mm isotropic) and are acquired before contrast injection.

A crucial component of this new technique is the myelin-sensitive RATIO image. Although more complex than the cortical rim with its sparsely myelinated outer and densely myelinated inner layers, the RATIO image also enhances the contrast differences between sparsely and densely myelinated or iron rich brainstem structures. Considering that the RATIO image is derived from the T1 and T2 images and has a superior gray/white contrast and myelin signal compared to these images, combining it with these two contrasts for the cluster analysis seems at first redundant. But as demonstrated here, combining the three images for the cluster analysis is an efficient way to reduce misclassification due to random noise and thus to achieve a better segmentation performance than it is possible with just the RATIO image.

The T1/T2 brainstem segmentation is based on a modification of a previously described segmentation approach (Mueller, 2020) that used the T1 relaxation map and T1 weighted image derived from the MP2Rage sequence (Marques et al., 2010) as input. The T1/T2 brainstem segmentation depicts the same structures as the MP2Rage based brainstem segmentation. The T1/T2 derived cluster maps primarily describing gray matter structures (cluster 3, cluster 5) and brainstem tissue/csf partial volume (cluster 2) look similar to the gray matter clusters generated with the MP2Rage approach, i.e., the same structures are grouped together in the T1/T2 and the MP2Rage segmentation. However, clusters 1 and 4 both depict white and gray matter structures together in one cluster but are separated by tissue class in the clusters generated by the MP2Rage approach. These mixed-tissue clusters cannot be explained by the different cluster number (6 clusters for the MP2Rage vs. 5 clusters for the T1/T2 approach) because clustering the intensities into 6 instead of 5 cluster number only further subdivides cluster 2 but keeps clusters 1 and 4 intact. There are two possible explanations for this: (1) In contrast to the T1/T2 approach, the images derived from the MP2Rage sequence do not have to be co-registered, i.e., the signal properties of the MP2Rage images are not affected by re-sampling and interpolation. (2) The T1/T2 signal is known to be influenced by other factors besides myelin (Arshad et al., 2017; Hagiwara et al., 2018; Uddin et al., 2018) and thus captures slightly different features than T1 or T2 relaxation based approaches. These differences suggest that the MP2Rage based approach might be superior to the T1/T2 based approach for the segmentation of internal brainstem structures. However, this needs to be confirmed by a direct comparison of the performance of these two approaches in the same population.

The majority of the investigated brainstem structures becomes smaller with age but the effect of age on their volume was modest as it explained <10% of the variation. The exception were VTA, SN, NR, CR, RPO, OI and NTS in which age explained between 10 to 20% of the volume variation. SN and NR are iron rich structures whose iron content increases with age (Keuken et al., 2017; Li et al., 2021). This not only enhances their contrast thus allowing for a more accurate segmentation but also causes a neuroinflammatory state and cell damage (Zucca et al., 2017) which could explain the more pronounced age-related volume loss in these nuclei. Age-related structural and functional alterations have also been described for the VTA (Siddiqi et al., 1999) NTS (Sturrock, 1992; Yamamoto et al., 2005; Hardy et al., 2018) and for the OI (Pesce et al., 1980; Sjöbeck et al., 1999; Lasn et al., 2006; Baizer et al., 2018) although the findings are less consistent than those for SN and NR. There exist to the best of our knowledge no studies investigating age effects in CR or RPO but functions in which these nuclei, play important roles i.e., locomotion, pain perception and sleep, are affected by age (Bassant and Poindessous-Jazat, 2002; Farrell, 2012; Lau et al., 2015) which supports the notion of age-related volume losses in these structures.

The age-related volume loss in brainstem gray matter structures found in this study complements the age-related myelin loss in brainstem white matter tracts described by Bouhrara et al. (2020) using myelin water fraction (MWF) mapping. Bouhrara et al. (2020) also tested for quadratic age – MWF associations and found that this model explained a higher percentage of the MWF variability in white matter of the midbrain and superior cerebral peduncle and the gray matter of the subthalamic nucleus, red nucleus and substantia nigra than linear age – MWF models. Testing for quadratic age – volume associations in this study identified significant associations for SN, RPT, RG, VLM, OR, right RMO, left CR and right Tld. However, the additional amount of variation explained by the quadratic model compared to the linear model was 1% or less which was consistent with the visual impression of a linear age-volume associations in these structures. The quadratic associations found by Bouhrara et al. (2020) were stronger. There are several possible explanations for this. The population studied by Bouhrara et al. (2020) included younger subjects and thus a larger age range, i.e., 21–94 years than this study with an age range 35–100 years. Lifetime white matter myelination follows an inverted U shape and peaks in the late twenties-early thirties (Bartzokis et al., 2004; Dvorak et al., 2021) which means that in contrast to Bouhrara et al. (2020) the myelination peak period is not adequately represented in this study. Furthermore, with the exception of SN, NR and subthalamic nucleus, Bouhrara et al. (2020) focused on well myelinated larger white matter structures and used MWF while this study focused on volumes of small and mostly sparsely myelinated gray matter structures.

This study has limitations. (1) The HCP-Aging project (ages 36–100 years) is part of the Human Connectome Lifespan project that also includes the HCP-Development project (ages 5–21 years). The HCP-Aging and HCP-Development use the same imaging protocol. The age range 21–35 years is covered by the HCP Young Adults project that used a slightly different imaging protocol, i.e., the T1 and T2 sequences have a higher resolution (0.7 mm isotropic instead of 0.8 mm isotropic) which complicates combining it with HCP-Aging and HCP-Development particularly when the focus of interest is small structures such as brainstem nuclei. (2) The re-scaling approach and in particular the reference values were taken from the MP2Rage approach. No attempts were made to optimize the reference values for the T1/T2 approach. Although the resulting segmentation quality was satisfactory, it cannot be excluded that optimizing these values would have improved the segmentation.

Taken together, the findings presented here suggest that it is possible to obtain a biologically meaningful segmentation of internal brainstem structures using a fully automated segmentation procedure and high resolution (0.8−1 mm isotropic) T1 and T2 weighted sequences. These sequences can be easily implemented into a clinical protocol. Combined with age corrected normal reference volumes, this technique could be used to screen for the type of subtle brainstem abnormalities that often precede the clinical manifestations of neurodegenerative diseases such as Alzheimer's and Parkinson's disease.

## Data availability statement

The original contributions presented in the study are included in the article/supplementary material, further inquiries can be directed to the corresponding author. The imaging data used in this publication is publicly available and comes from the Human Connectome Project (HCP) Aging in the NDA data repository (https://nda.nih.gov/). The Matlab scripts for the brainstem segmentation described here are available at https://data.mendeley.com/datasets/8pfjgrwtsr/1.

## Ethics statement

The requirement of ethical approval was waived by the University of California, San Francisco, IRB for the studies involving humans because Data from the Human Connectome Aging project (HCP) was used for this study. The data is completely de-identified. Studies using anonymized data where it is not possible to ascertain the subject's identity are considered nonhuman subject studies and exempt from UCSF IRB review.

## Author contributions

SM: Conceptualization, Formal analysis, Methodology, Writing—original draft, Writing—review & editing.
